# Helpers at the Nest Improve Late-Life Offspring Performance: Evidence from a Long-Term Study and a Cross-Foster Experiment

**DOI:** 10.1371/journal.pone.0033167

**Published:** 2012-04-04

**Authors:** Lyanne Brouwer, David S. Richardson, Jan Komdeur

**Affiliations:** 1 Animal Ecology group, Centre for Ecological and Evolutionary Studies, University of Groningen, Groningen, The Netherlands; 2 Evolution, Ecology and Genetics, Research School of Biology, The Australian National University, Canberra, Australia; 3 Centre for Ecology, Evolution and Conservation, School of Biological Sciences, University of East Anglia, Norwich, United Kingdom; 4 Nature Seychelles, Mahé, Republic of Seychelles; 5 Behavioural Ecology and Self-organisation, Centre for Ecological and Evolutionary Studies, University of Groningen, Groningen, The Netherlands; University of Manitoba, Canada

## Abstract

**Background:**

Conditions during an individual's rearing period can have far reaching consequences for its survival and reproduction later in life. Conditions typically vary due to variation in parental quality and/or the environment, but in cooperative breeders the presence of helpers adds an important component to this. Determining the causal effect of helpers on offspring fitness is difficult, since high-quality breeders or territories are likely to produce high-quality offspring, but are also more likely to have helpers because of past reproductive success. This problem is best resolved by comparing the effect of both helping and non-helping subordinates on offspring fitness, however species in which both type of subordinates commonly occur are rare.

**Methodology/Principal Findings:**

We used multi-state capture-recapture models on 20 years of data to investigate the effect of rearing conditions on survival and recruitment in the cooperatively breeding Seychelles warbler (*Acrocephalus sechellensis*), with both helping and non-helping subordinates. The number of helpers in the rearing territory, but not territory quality, group- or brood size, was positively associated with survival of offspring in their first year, and later in life. This was not a result of group size itself since the number of non-helpers was not associated with offspring survival. Furthermore, a nestling cross-foster experiment showed that the number of helpers on the pre-foster territory was not associated with offspring survival, indicating that offspring from territories with helpers do not differ in (genetic) quality.

**Conclusions/Significance:**

Our results suggest that the presence of helpers not only increase survival of offspring in their first year of life, but also subsequent adult survival, and therefore have important fitness consequences later in life. This means that when calculating the fitness benefits of helping not only short-term but also the late-life benefits have to be taken into account to fully understand the evolution of cooperative breeding.

## Introduction

The conditions that individuals experience during the rearing period can vary due to differences in the environment and/or of the parents. Environmental effects can differ between individuals because of variation in, for example, territory quality [1], or can affect entire cohorts, for example due to bad weather in a specific year [2], [3]. Parents can affect the quality of offspring directly via the genes that the offspring inherit, but also as a result of their reproductive decisions, e.g. the trade-off between the quantity and quality of offspring [4], [5] or current and future reproduction [6]. Parents can also contribute to how the environment affects early development [7], since parents can increase provisioning when resource availability is low [8] or adjust egg size or composition which can affect offspring growth, survival or immune function [9]–[11].

Until recently, it was thought that the variation in conditions that individuals experience during the rearing period would only have short-term effects on fitness components early in life. Effects of rearing conditions on fitness components later in life were expected to be overridden by environmental stochasticity accumulating during individual's lifetime [1], and because selection on fitness components becomes weaker over the course of life [12], [13]. However, the long-term consequences of rearing conditions have now become clear [7], [14], [15] and numerous studies have shown that such conditions can have important fitness consequences later in life [1], [16]–[20]. Although the evidence for long-term fitness consequences of conditions during early development in long-lived birds is still debated (for review see: [21]).

In cooperatively breeding species, individuals delay dispersal and often help to rear kin [22]. Helpers add an important component to rearing conditions as their helping behaviour has been shown to positively affect offspring survival and body weight early in life [23], [24]. Recently a number of studies have shown that helpers can have long-term benefits for the helped offspring through improving survival to maturity [25]–[27], advancing the onset of first reproduction [25], [28] or increasing lifetime reproductive success [29]. However, in long-lived species variation in lifetime fitness is best explained by the number of breeding attempts (and thus adult longevity/survival) rather than by individual differences in annual reproductive output [30], [31]. Yet, whether the presence of helpers can even affect adult survival of the helped offspring remains unknown. Positive effects of helpers on offspring performance are essential when explaining cooperative breeding through kin-selection [23], [32] or group-augmentation [33]. If helpers also affect offspring performance later in life then the calculations based on the short-term benefits will be an underestimation of fitness benefits of helping and thereby complicate our understanding on the evolution of cooperative breeding.

An important difficulty in interpreting how variation in rearing conditions affects fitness is the inability to distinguish rearing effects from individual quality effects, i.e. high-quality parents might occupy high-quality territories and produce high-quality offspring, which survive better. Cross-foster experiments, in which nestlings are swapped between nests, are able to resolve this problem by separating the rearing from the (genetic) quality effects, although cross-fostering does not separate the individual (genetic) quality from territory quality. In cooperative breeders, high-quality breeders/territories are also likely to recruit more helpers because of past breeding success, resulting in, possibly pervasive, non-causal correlations between the presence of helpers and offspring fitness [22], [34]. To establish causality, the effect of helping has to be distinguished from the fact that living in a high-quality territory or a larger group (‘group augmentation’, [33]) can be beneficial itself.

Several approaches have been suggested to determine causality of helping. First, experiments in which helpers were removed have shown that offspring perform less well after helper removal [35]–[37], however these experiments potentially disrupt social relationships within the group [36]. Second, a comparison of the same group with and without helpers has been suggested to determine the causality of helper effects [38], [39]. Such comparisons have been criticized as groups where helper numbers change might be a biased sample of the population [34], as changes in helper number are the result of high reproduction or low survival. However a recent study using this approach suggests that this criticism is not necessarily valid [40]. Third, it has been suggested that statistical models that incorporate the effect of territory or breeder identity as random effects may disentangle helper from quality effects [22]. However, disentangling and reliably estimating such variance components typically requires large sample sizes as well as biological factors alleviating any covariance between individual and territory quality (e.g. by breeders switching territories) [40]. Fourth, a very powerful method is to compare offspring from groups where subordinates provision with offspring from groups where subordinates do not help [41], [42], but such an approach is only applicable in species where subordinates often fail to provision, which is rare among cooperative breeders [40].

Variation in rearing conditions can also affect natal dispersal patterns, for example birds of high phenotypic quality disperse when they are born in low quality habitat [43]. An important methodological consequence of such a biological phenomenon is that in studies that concern open infinite populations, unobserved dispersal outside the study population (permanent emigration) will be erroneously interpreted as mortality, and consequently effects of rearing conditions on dispersal and survival are confounded [44]–[46]. However, the effects of rearing conditions on survival and recruitment can be unambiguously determined in closed populations (i.e. no emigration).

Using multistate mark-recapture analyses on 20 years of data we investigate the effect of rearing conditions on juvenile and adult survival and recruitment of Seychelles warbler (*Acrocephalus sechellensis*) offspring. This cooperative breeder is endemic to a few small islands in the Indian Ocean. The population on Cousin Island is a closed population, since dispersal from the island is virtually absent [47]. Although Seychelles warblers can breed independently in their first year, a lack of suitable habitat drives some males as well as females to become subordinate within their natal territory [48], [49]. Nestlings are fed for up to three months and remain in the natal territory for at least six months [50], suggesting helpers in the natal territory have ample opportunity to make substantial improvements to early life conditions of offspring. A subordinates' decision to help is independent of territory quality (measured according to Komdeur [48]), and for female subordinates has been shown to depend on the continued presence of the primary female that raised them (the putative mother), thus assuring they gain kin-selected benefits through helping [51], [52]. A helper removal experiment has shown that helping increased reproductive success of Seychelles warblers by increasing nestling survival [53], but it is unknown whether helpers also have long-lasting positive effects on offspring fitness.

Here we investigate the long-term effect of conditions during the rearing period upon subsequent juvenile and adult survival and the probability of being recruited into a breeding position. We considered territory quality, group size, brood size and the number of helping and non-helping subordinates in the rearing territory as potential key aspects of conditions during the rearing period. Previous analyses have shown that natal territory quality and natal group size do not affect juvenile survival [54]. We first explore the association between rearing conditions and offspring survival and recruitment. By investigating both the number of helping and non-helping subordinates we will be able to test whether associations are due to causal effects of helping or correlated effects through group size. Furthermore, we will use data from a cross-fostering experiment of nestlings [55] to distinguish rearing from genetic (quality) effects. We do this by comparing the effects of the conditions in the original (pre-foster) territories to those of the rearing territories. If any effects of rearing conditions on survival or recruitment are the result of a causal relationship, we would expect an association between conditions of the rearing territories, and not of the original territories, on the performance of cross-fostered offspring.

## Methods

### Ethics statement

The work has been conducted under the proper legislation of the Seychelles law; the Department of Environment and the Seychelles Bureau of Standards gave permission for fieldwork and sampling (approval reference A0347). Our work also complied with all the ethical conditions set out by the European institutions involved (University of Groningen & University of East Anglia).

### Study area and data collection

Data were collected as part of the long term study of the Seychelles warbler population on Cousin Island (04°20′S, 55°40′E) from 1986 to 2006 [55], [56]. See Table 1 for a summary of years in which specific data was collected. During the main breeding season (July to September), and in some years during the minor breeding peak (January to March), each territory was checked for the presence of birds and breeding activity [48], [54]. We assigned the status of all birds in the population. The ‘primary’ male and female were defined as the pair-bonded male and female in the territory. All other adult birds resident in the territory were defined as ‘subordinate’ [57]. Nests were observed throughout the breeding cycle. Most Seychelles warblers produce one clutch per season and this normally consists of just one egg, but about 20% of nests contain two or three eggs [58]. Parentage analysis has shown that egg dumping does not occur, however, joint-nesting is common with 44% of subordinate females producing offspring. Moreover, 40% of offspring are the result of extra-group paternity (sired by a male from outside the social group) [58]. Birds were either ringed as nestlings or as fledglings while still resident in the natal territory and dependent on their parents (birds of known age and origin), or later when independent (birds of unknown age and origin). Birds were ringed with a unique combination of three colour rings and a British Trust for Ornithology ring and since 1993 all birds were blood-sampled.

**Table 1:** Summary showing the years in which specific data was collected to investigate the effects of early conditions on survival of Seychelles warblers between 1986–2006.

Data collected	Year
Mark–recapture/resighting main breeding season	1986–1991, 1993–2006
Mark–recapture/resighting minor breeding season	1998, 1999, 2004, 2005
DNA sexing	1993–2006
Territory quality	1987, 1990, 1996–1999, 2003–2006
Group size	1986–1991, 1993–2006
No. helpers	1997–1999, 2002–2005
No. non-helpers	1997–1999, 2002–2005
Brood size	1997–1999, 2002–2005
Nestling cross-fostering	1997–1999

Molecular sexing [59] was used to determine the sex of each individual sampled since 1993. Before that birds were sexed based on observations and biometry at 6 months of age [60]. Consequently, by including only birds of known sex, juvenile survival before 1993 will likely be overestimated (as for the earlier period it will only include birds that reached 6 months of age). However, here we are interested in the long-term effects of rearing conditions, i.e. their effect on adult survival and recruitment.

### Operationalization of rearing conditions

Rearing conditions were defined as the conditions (i.e. group size, territory quality etc.) in the rearing territory during the breeding season the bird hatched. Group size is defined as the number of independent birds resident in the territory. Seychelles warblers are insectivorous, taking 98% of their insect food from leaves, therefore an index of territory quality was calculated using the number of insect prey available, territory size and foliage cover following the methods in Komdeur [48]. Territory quality was calculated for each territory in 1987, 1990, 1996–1999 and 2003–2006. The territories are very static in space (Brouwer, Richardson & Komdeur pers. obs.) and the number of territories varies little over time (number of territories: 112.3±1.2 S.E., *n* = 15 years). Consequently, for the remaining years, territory quality for each territory was calculated as the average from the preceding and following period [54].

Additional data on rearing conditions was available for the cohorts 1997–1999 and 2002–2005 (*n* = 327). For these cohorts brood size was available and group size was specified as the number of helping and non-helping subordinates. During the nestling provisioning stage a minimum of two 90-minute observations (1 week apart and randomized with respect to time of day) were completed at each nest to asses whether a subordinate was helping or not [49]. A subordinate was defined as ‘helper’ when it provisioned nestlings whereas ‘non-helpers’ were never observed provisioning. Females and males are as likely to act as helper [61], but joint nesting females (female subordinates laying an egg) might have wrongly been assigned as helper in this study (since a full pedigree is not yet available). However, our main interest lies in how additional helping behaviour affects offspring fitness.

### Cross-foster experiment

To disentangle the effects of rearing conditions and (genetic) quality on long-term fitness, we investigated survival and recruitment of 69 same age (±2-day difference) nestlings that were cross-fostered between 0 and 6 days of age for the cohorts 1997–1999. Nests with two or three nestlings (*n* = 11) were reduced to one by moving two or three nestlings to another nest, but provisioning rates have been shown to be independent of the number of nestlings [51].

### Survival and recruitment analysis

**Figure 1:**
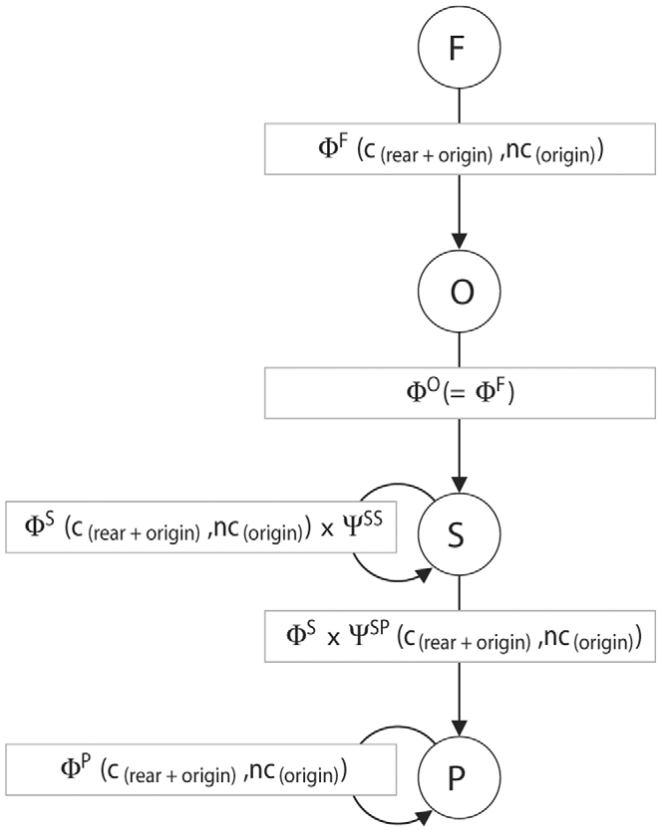
Schematic overview of the life cycle of the Seychelles warbler. The four main life stages: fledgling (F), old fledgling (O), subordinate (S) and primary (P) with the survival (Φ) and transition (Ψ) parameters as estimated in the multistate capture-recapture model as a function of covariates of the rearing and original (pre-foster) territory. (c) = cross-fostered, (nc) = non-cross-fostered. After the first year of life ‘old fledgling’ birds subsequently become ‘subordinate’, recruit to a ‘primary’ (breeding) position, or die. The transition probabilities from fledgling to old fledgling, and from old fledgling to subordinate were fixed to one, as all fledglings and old fledglings move to the next state, conditional that they survive.

We constructed the capture-resighting histories of 1047 marked birds that were monitored between 1986 and 2006. Of these, 499 were ringed as nestling or fledgling (known age and territory). Although birds ringed as adults do not provide any direct information about effects of rearing conditions, they were included in the analyses to improve the estimation of parameters that were independent of rearing conditions and thereby indirectly improve the accuracy of parameters of key interest. The capture-resighting histories were included in one combined model, using multistate mark-recapture models based on resightings (e.g. [62]). Survival, resighting and transition probabilities between the states of ‘fledgling’, ‘old fledgling’, ‘subordinate’ and ‘primary’ were estimated according to Figure 1. Since offspring have never been observed to help or breed before six months of age [50] they can be considered as juveniles in two consecutive periods in years where both the major and minor breeding peaks are monitored. To include this in the multistate model, the first year of life was divided into two states: ‘fledgling’ and ‘old fledgling’. After the first year of life ‘old fledgling’ birds subsequently become ‘subordinate’, recruit to a ‘primary’ (breeding) position, or die. The transition probabilities from fledgling to old fledgling, and from old fledgling to subordinate were fixed to one, as all fledglings and old fledglings move to the next state, conditional that they survive (Figure 1). Twenty-nine out of 1047 birds in our dataset lost their ‘primary’ status and became ‘subordinate’ again. Although this is an interesting phenomenon [63], we did not include these birds in our sample as we are primarily interested in recruitment here. Consequently, the transition from ‘primary’ to ‘subordinate’ was constrained to zero (Figure 1).

Each year, except for 1992, individuals were recorded as present if observed in the last two weeks of the main breeding season (1 July–1 September). Furthermore, for 1998, 1999, 2004 and 2005 the minor breeding peak (1 January–1 March) was monitored. The biannual re-sighting periods allow us to estimate survival over two 6-month periods, for the remaining years we could only calculate survival over the whole year. If no capture-resighting data were available for the minor breeding season, dummy variables were created by including zeros in the encounter histories and adjusting the time interval, with the survival parameter (Φ) set to 1 and the resighting parameter (recapture, *p*) and transition parameter (ψ) set to 0 [54]. A total of 58 birds that were translocated in 2004 [56] were removed from the dataset from that moment on (i.e. treated as right censored). Individuals' re-sighting histories were used as input files for survival analyses in the program MARK [64].

We employed an *a priori* approach in which a small set of candidate models was created based on previous knowledge and hypotheses of interest. Previous analyses have shown that annual survival was high, both for juveniles (first year) (0.61) and adults (0.84), and did not differ between the sexes [54]. The basic model structure we use here (Table 2, model 3) allowed survival to vary between years and states, with different survival probabilities for individuals in their first year of life (fledgling and old fledgling state) than for older birds (subordinate and primary state). We expected the resighting probability to be highest for primary birds, because they remain in the same territory after settling, in contrast to subordinates which make forays around the island in search for a vacancy [65]. To simplify and avoid the over-parameterisation of our model we assumed time-independent resighting rates but allowed them to vary between the primary and the other states. Our basic model structure allowed transition (recruitment) probabilities to vary over time and between the sexes. In addition, three groups were created in the analyses, one group for birds of known age and origin, one group for cross-fostered (also known age and origin) offspring, and one group for the birds of unknown age and origin. For each year that data was available, conditions of the rearing territory were included as individual covariates in the analyses for both the cross-fostered birds and for birds of known age and origin. However, the individual covariates describing the conditions of the original territory (pre-fostering) were included for the cross-fostered birds only. Since previous analyses have shown that local density negatively affected adult survival [54], the average group size a bird lived in from its second year on was included as a covariate on adult survival when testing for the effects of the rearing conditions.

**Table 2:** Results of a multistate model examining survival (Φ), resighting (*p*) and recruitment probabilities (transition from subordinate to primary state, Ψ*^SP^*) for Seychelles warblers (*n* = 1018) from 1986 to 2006.

No.	Model	Description of effect	No. Par.	ΔQAIC_c_	QAIC_c_ weights
1	Φ*^F^* ^O^_(t)≠_Φ*^S^*_ = _Φ*^P^*_(t)_ *p^FO^*_(.) = _ *p^S^*_(.)≠_ *p^P^*_(.)_ψ*^SP^*_(t)_	Absence of sex effect on recruitment	67	0.0	0.74
2	Φ*^FO^*_(t)≠_Φ*^S^*_ = _Φ*^P^*_(t)_ *p^FO^*_(.) = _ *p^S^*_(.)≠_ *p^P^*_(.)_ ψ*^SP^*_(t+s)_	Additive effect of sex on resighting	68	2.1	0.26
3	Φ*^FO^*_(t)≠_Φ*^S^*_ = _Φ*^P^*_(t)_ *p^FO^*_(.) = _ *p^S^*_(.)≠_ *p^P^*_(.)_ψ*^SP^*_(t×s)_	Starting model	84	17.0	0.00
4	Φ*^FO^*_(t)≠_Φ*^S^*_(t)≠_Φ*^P^*_(t)_ *p^FO^*_(.) = _ *p^S^*_(.)≠_ *p^P^*_(.)_ψ*^SP^*_(t×s)_	Survival function of state	106	46.3	0.00
5	Φ*^FO^*_(t)≠_Φ*^S^*_(t)≠_Φ*^P^*_(t)_ *p^FO^*_(.)≠_ *p^S^*_(.)≠_ *p^P^*_(.)_ψ*^SP^*_(t×s)_	Survival and resighting function of state	107	47.9	0.00

The different states (life stages) are: fledgling (F), old fledgling (O), subordinate (S) and primary (P). (t) = time, (s) = sex, (.) = constant. Models were ranked according to their QAIC_c_ value, with the best supported model on top. ΔQAIC_c_ being the difference between the QAIC_c_ of the best supported model and the model considered.

We first investigated whether the probability of resighting or survival varied between birds in the different states and checked whether the recruitment rate differed between the sexes. We then investigated whether survival and recruitment were associated with conditions during the rearing period for both the cross-fostered birds and the other birds of known age and origin. Finally, we investigated whether we can distinguish rearing conditions from (genetic) quality effects by including conditions of the original (pre-fostering) territory for cross-fostered offspring only.

Model selection was based on Akaike's Information Criterion (AICc) corrected for sample size with better fitting models resulting in lower AIC_c_ values [66], but models with ΔAICc <2 are considered to be approximately equally well supported. Additionally, we report the normalized Akaike weights to assess the relative support for competing models [67]. Estimating the amount of overdispersion using the median ĉ-procedure implemented in program MARK [68] showed some evidence for overdispersion (variance inflation factor *ĉ* = 1.51±0.02). Consequently, AIC_c_ values were adjusted to allow for the extent of overdispersion measured by *ĉ*, through quasi likelihood (QAIC_c_).

## Results

### Natural variation in rearing conditions

On average 39% of the territories (average total number of territories on Cousin Island = 112.3±4.6 S.D.) had one or more subordinates in a given year. The nest observation data showed that group sizes varied from 2 to 6 birds, with a maximum of two helping (mean = 0.29±0.52 S.D.) and two non-helping (mean = 0.53±0.70 S.D.) subordinates observed per territory (groups of 7 birds exist but are rare and were not part of this dataset). The index of territory quality revealed that higher quality territories did not have more helpers, nor non-helpers, than lower quality territories (GLMM, response variable territory quality with territory identity as random effect, *n* = 1117, helpers: 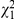 = 0.87, *P* = 0.35; non-helpers, 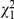 = 0.31, *P* = 0.58). However, there could be other aspects associated with the presence of subordinates not accounted for in the territory quality calculation.

### State, sex and time-dependent variation

We investigated whether survival, recruitment and resighting probabilities differed between individuals in the different states (Figure 1). Annual resighting probabilities were similarly high for first year birds (fledglings and old fledglings) as subordinates (0.83±0.02 S.E.), but even higher for primary birds (0.97±0.01 S.E.; Table 2, model 3 vs. 5). There was no evidence that survival probabilities differed between subordinates and primaries (Table 2, model 3 vs. 4). Annual recruitment probabilities varied between 0.15 and 0.79 (average = 0.60±0.04 S.E.). There was no evidence for differential recruitment between the sexes (Table 2, model 1 vs. 2; *β* = −0.002±0.21), and this did not change between years (Table 2, model 1 vs. 3). A model with equal recruitment probabilities for both sexes was 2.8 times better supported by the data than a model with sex-specific recruitment (Table 2, model 1 vs. 2). Consequently model 1 (Table 2) was used as a starting model to investigate the effects of conditions during the rearing period on survival and recruitment.

### Effect of rearing conditions on survival

**Figure 2:**
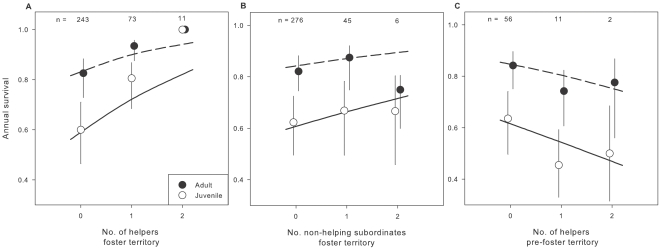
Annual adult and juvenile survival of Seychelles warblers. Survival probabilities (with S.E.) and model predictions for an average year are given in relation to a) the number of helpers in the rearing territory (predictions based on model 1, Table 3a), b) the number of non-helpers in the rearing territory (predictions based on model 5, Table 3a) and c) the number of helpers in the original (pre-foster) territory of cross-fostered offspring (predictions based on model 3, Table 4a). Numbers on top indicate number of offspring followed.

We found that the number of helpers in the rearing territory (of both cross-fostered and non-cross fostered offspring) was positively associated with survival (Table 3a, model 1 vs. 3), and that this effect did not vary between years (Table 3a, model 11) or with territory quality (Table 3a, model 4 vs. 1). Specifically, the number of helpers in the rearing territory was not only positively associated with survival in the first year of life but also later in life, as there was no evidence that the effect varied between the fledgling/old fledgling state versus the subordinate and primary states (Table 3a, model 1 vs. 2; Figure 2a). Including the number of helpers in the rearing territory as a covariate with survival was 3.8 times better supported by the data than a model without this effect (Table 3a, model 1 vs. 3). This effect was due to the presence of helpers itself as there was no evidence that the number of non-helping subordinates (Table 3a, models 5 and 8 vs. 3; Figure 2b), or group size (helping and non-helping subordinates; Table 3a, model 10 vs. 3) in the rearing territory was associated with juvenile or adult survival. Including the number of non-helping subordinates as a quadratic effect did not improve the fit of the model (Table 3a, model 7 vs. 5). Brood size and territory quality of the rearing territory were also not associated with survival (Table 3a, models 6 and 9 vs. 3).

**Table 3:** Results of a multistate model examining conditions during the rearing period on (a) survival (Φ) and (b) recruitment (transition from subordinate to primary state, Ψ*^SP^*) of Seychelles warblers.

No.	Model	Description of effect	No. Par.	ΔQAIC_c_	QAIC_c_ weight
(a)					
1	(Φ*^FO^*_≠_Φ*^S^*_ = _Φ*^P^*) _t+h_ ψ*^SP^*_(t)_	Helper on survival	69	0.0	0.45
2	Φ*^FO^*_(t+h)≠_Φ*^S^*_ = _Φ*^P^*_(t+h)_ ψ*^SP^*_(t)_	Helper on survival in interaction with state (juvenile vs. adult)	70	2.0	0.16
3	Φ*^FO^*_(t)≠_Φ*^S^*_ = _Φ*^P^*_(t)_ ψ*^SP^*_(t)_	Starting model	68	2.7	0.12
4	(Φ*^FO^*_≠_Φ*^S^*_ = _Φ*^P^*) _t+(h×tq)_ ψ*^SP^*_(t)_	Helper in interaction with territory quality on survival	71	3.2	0.09
5	(Φ*^FO^*_≠_Φ*^S^*_ = _Φ*^P^*) _t+nh_ ψ*^SP^*_(t)_	Non-helper on survival	69	4.0	0.06
6	Φ*^FO^*_(t+tq)≠_Φ*^S^*_ = _Φ*^P^*_(t+tq)_ ψ*^SP^*_(t)_	Territory quality on survival in interaction with state (juvenile. vs. adult)	70	5.6	0.03
7	(Φ*^FO^*_≠_Φ*^S^*_ = _Φ*^P^*) _t+(nh)_^2^ ψ*^SP^*_(t)_	Quadratic effect non-helper on survival	70	5.8	0.02
8	Φ*^FO^*_(t+nh)≠_Φ*^S^*_ = _Φ*^P^*_(t+nh)_ ψ*^SP^*_(t)_	Non-helper on survival in interaction with state (juvenile vs. adult)	70	6.0	0.02
9	Φ*^FO^*_(t+b)≠_Φ*^S^*_ = _Φ*^P^*_(t+b)_ ψ*^SP^*_(t)_	Brood size on survival in interaction with state (juvenile vs. adult)	70	6.2	0.02
10	Φ*^FO^*_(t+gs)≠_Φ*^S^*_ = _Φ*^P^*_(t+gs)_ ψ*^SP^*_(t)_	Group size on survival in interaction with state (juvenile vs. adult)	70	6.5	0.02
11	(Φ*^FO^*_≠_Φ*^S^*_ = _Φ*^P^*)_(t×h)_ ψ*^SP^*_(t)_	Time dependent helper on survival	72	23.6	<0.01
(b)					
1	Φ*^FO^*_(t)≠_Φ*^S^*_ = _Φ*^P^*_(t)_ ψ*^SP^*_(t)_	Starting model	68	0.0	0.30
2	Φ*^FO^*_(t)≠_Φ*^S^*_ = _Φ*^P^*_(t)_ ψ*^SP^*_(t+b)_	Brood size on recruitment	69	0.5	0.23
3	Φ*^FO^*_(t)≠_Φ*^S^*_ = _Φ*^P^*_(t)_ ψ*^SP^*_(t+h)_	Helper on recruitment	69	1.8	0.12
4	Φ*^FO^*_(t)≠_Φ*^S^*_ = _Φ*^P^*_(t)_ ψ*^SP^*_(t+gs)_	Group size on recruitment	69	1.9	0.12
5	Φ*^FO^*_(t)≠_Φ*^S^*_ = _Φ*^P^*_(t)_ ψ*^SP^*_(t+tq)_	Territory quality on recruitment	69	1.9	0.12
6	Φ*^FO^*_(t)≠_Φ*^S^*_ = _Φ*^P^*_(t)_ ψ*^SP^*_(t+nh)_	Non-helper on recruitment	69	2.0	0.11

The different states (life stages) are: fledgling (F), old fledgling (O), subordinate (S) and primary (P). Covariates of the rearing territory: (h) = number of helpers, (nh) = number of non-helpers, (tq) = territory quality, (b) = brood size, (gs) = group size, (t) = time. Models were ranked according to their QAIC_c_ value, with the best supported model on top. ΔQAIC_c_ being the difference between the QAIC_c_ of the best supported model and the model considered.

### Effect of rearing conditions on recruitment

The probability of recruitment to the primary state was not higher for birds reared on a territory with helpers (Table 3b, model 3 vs. model 1). Furthermore, recruitment probabilities were not associated with territory quality, the number of non-helpers or the size of the group or brood in which they were reared (Table 3b).

### Disentangling rearing from (genetic) quality effects on survival and recruitment

The association between the number of helpers in the territory and offspring survival was not a result of a non-causal relationship caused by (genetic) quality; the cross-foster experiment showed that the number of helpers of the original (pre-foster) territory was not associated with either survival (Table 4a, model 3 vs. 1) or recruitment probabilities (Table 4b, model 1 vs. 2). Furthermore, none of the other characteristics of the original territory i.e. group size, territory quality, brood size and the number of non-helpers were associated with survival or recruitment of the cross-fostered offspring (Table 4). This null-result was not likely caused by a lack of power as there was a positive association between the number of helpers in the foster territory and both juvenile and adult survival for the cross-fostered offspring (Table 4a, model 1 vs. 2). Although QAIC_c_ increased by only 1.8, including the number of helpers in the foster territory was 2.4 times better supported by the data than a model without this effect (Table 4a, model 1 vs. 2).

**Table 4:** Results of a multistate model examining the effects of the original (pre-foster) territory conditions on (a) survival and (b) recruitment probabilities (transition from subordinate to primary state, ψ*^SP^*) of cross-fostered Seychelles warblers (n = 69).

No.	Model	Description of effect	No. Par.	ΔQAIC_c_	QAIC_c_ weights
(a)					
1	(Φ*^FO^*_≠_Φ*^S^*_ = _Φ*^P^*) _(t+fosterh)_ ψ*^SP^*_(t)_	Helper foster territory on survival	69	0.0	0.50
2	Φ*^FO^*_(t)≠_Φ*^S^*_ = _Φ*^P^*_(t)_ ψ*^SP^*_(t)_	Starting model	68	1.8	0.20
3	(Φ*^FO^*_≠_Φ*^S^*_ = _Φ*^P^*) _(t+h)_ ψ*^SP^*_(t)_	Helper original territory on survival	69	3.8	0.08
4	(Φ*^FO^*_≠_Φ*^S^*_ = _Φ*^P^*) _(t+h)_^2^ ψ*^SP^*_(t)_	Quadratic effect helper original territory on survival	70	3.9	0.07
5	Φ*^FO^*_(t+nh)≠_Φ*^S^*_ = _Φ*^P^*_(t+nh)_ ψ*^SP^*_(t)_	Non-helper original territory on survival in interaction with state (juvenile vs. adult)	70	4.1	0.06
6	Φ*^FO^*_(t+b)≠_Φ*^S^*_ = _Φ*^P^*_(t+b)_ ψ*^SP^*_(t)_	Brood size original territory on survival in interaction with state (juvenile vs. adult)	70	5.4	0.03
7	Φ*^FO^*_(t+tq)≠_Φ*^S^*_ = _Φ*^P^*_(t+tq)_ ψ*^SP^*_(t)_	Quality original territory on survival in interaction with state (juvenile vs. adult)	70	5.5	0.03
8	Φ*^FO^*_(t+gs)≠_Φ*^S^*_ = _Φ*^P^*_(t+gs)_ ψ*^SP^*_(t)_	Group size original territory on survival in interaction with state (juvenile vs. adult)	70	5.7	0.03
(b)					
1	Φ*^FO^*_(t)≠_Φ*^S^*_ = _Φ*^P^*_(t)_ ψ*^SP^*_(t+h)_	Helper original territory on recruitment	69	0.0	0.27
2	Φ*^FO^*_(t)≠_Φ*^S^*_ = _Φ*^P^*_(t)_ ψ*^SP^*_(t)_	Starting model	68	0.5	0.21
3	Φ*^FO^*_(t)≠_Φ*^S^*_ = _Φ*^P^*_(t)_ ψ*^SP^*_(t+b)_	Brood size original territory on recruitment	69	0.6	0.20
4	Φ*^FO^*_(t)≠_Φ*^S^*_ = _Φ*^P^*_(t)_ ψ*^SP^*_(t+gs)_	Group size original territory on recruitment	69	1.4	0.14
5	Φ*^FO^*_(t)≠_Φ*^S^*_ = _Φ*^P^*_(t)_ ψ*^SP^*_(t+tq)_	Quality original territory on recruitment	69	1.9	0.10
6	Φ*^FO^*_(t)≠_Φ*^S^*_ = _Φ*^P^*_(t)_ ψ*^SP^*_(t+nh)_	Non-helpers original territory	69	2.5	0.08

Models were based on all individuals (n = 1018) but the covariates were included for the cross-fostered offspring only. The different states (life stages) are: fledgling (F), old fledgling (O), subordinate (S) and primary (P). Covariates from original territories: (h) = number of helpers, (nh) = number of non-helpers, (tq) = territory quality, (b) = brood size, (gs) = group size, (t) = time and (fosterh) = number of helpers on foster territory. Models were ranked according to their QAIC_c_ value, with the best supported model on top. ΔQAIC_c_ being the difference between the QAIC_c_ of the best supported model and the model considered.

## Discussion

Our results suggest that the presence of helpers has long-term effects on the offspring they help; the number of helpers in the rearing territory was not only associated with juvenile survival, but also with the later adult survival of the helped offspring. The evidence suggest that offspring benefited from being helped, rather than just the presence of other group members, as the number of non-helping subordinates was not associated with survival. Furthermore, the positive association between helper numbers and survival was not a non-causal result of offspring of groups with helpers being of higher (genetic) quality since the presence of helpers on the original (pre-foster) territory was not associated with offspring survival. These findings mean that not only the short-term but also the long-term benefits have to be taken into account to fully understand the evolution of cooperative breeding. Potentially other factors not accounted for here, like age and breeding experience, differ between groups with helpers versus groups with non-helping subordinates and result in differences in offspring survival. Although previous work has shown that age and breeding experience do affect reproductive success, access to food and foraging efficiency has been shown to be similar between older/more experienced and younger birds [69], [70]. It is therefore unlikely that age or breeding experience caused differences in survival after fledging.

Evidence that helpers can have short-term effects on offspring fitness by increasing juvenile survival has previously been shown, for example through removal experiments in several cooperatively breeding species [35]–[37], [71]. However whether helping has long-term fitness benefits has long remained unclear, reflecting the fact that helper effects are hard to study, not only because long-term data are needed, but also since specific approaches (e.g. comparing helping and non-helping subordinates) are required to determine the direction of causation [22]. Furthermore, parents might respond to the presence of helpers by reducing their investment in their offspring, making it even harder to detect helper effects as shown, for example, in a study on superb-fairy wrens (*Malurus cyaneus*) where mothers breeding in the presence of helpers lay smaller eggs of lower nutritional content that produce lighter chicks, as compared with those laying eggs in the absence of helpers [72]. In studies that have shown long-term effects of helping the benefit was mediated through increased mass or size at independence, which resulted in higher survival to maturity or younger age of first reproduction [25]–[29]. We have now shown that helping can even affect the adult survival of the helped offspring. It is likely, and logical, that the increased survival of Seychelles warblers may also be linked to higher body mass of the helped offspring at fledging as a result of the provisioning provided by helpers. However, helpers could also reduce the need for offspring to forage, which in turn could lead to reduced physiological damage like reduced oxidative stress [73], something that will be investigated in the future.

In the Seychelles warbler, the presence of helpers during the rearing period positively affects survival during the nestling stage [53], and also later in life, but did not result in a higher likelihood of recruiting to a breeder position (this study). A removal experiment indeed showed that the proximity to a vacant breeding position, rather than body size, was the main determinant of the chance of claiming a vacancy [74]. Nevertheless, since many individuals will not obtain a breeding position upon reaching adulthood, increasing one's adult survival will also increase one's likelihood of reproduction via joint-laying or extra-pair paternity as well as eventually obtaining a breeding vacancy. Although having helpers in a group therefore seems very beneficial for the future survival of the offspring, living in larger groups later in life becomes a disadvantage as this results in lower survival [54], most likely acting via competition for food [56]. This might explain why the presence of more than one helper is uncommon in this species (average no. of helpers per territory = 0.29±0.52 S.D.).

Neither brood size nor territory quality experienced during the rearing period was associated with survival at any stage of an individuals' life. A previous study on the Seychelles warbler has already shown that although there was variation in juvenile survival between seasons, this did not affect a cohorts' adult survival probability [54]. The long period in which the Seychelles warbler offspring are dependent on their parents might counterbalance any negative effects experienced during early life. Our results suggest that there is a direct effect of the additional care, probably as a result of the extra provisioning gained by young. Furthermore, a previous analysis found evidence for maternal effects, as maternal heterozygosity at microsatellite loci was positively associated with offspring survival [55], [75]. It is possible that parental effects are, therefore, a more important source of variation in quality than the effects of the environment and territory. The Seychelles warbler lives in a relatively stable tropical environment and birds time their reproduction to periods with high food availability and choose whether to lay one or two eggs [76]. With such a strategy, adverse conditions might be avoided. Although studies on temperate species might show greater effects of rearing conditions, in tropical species with less variation in the environmental conditions, parental effects and decisions might be more important.

Primary birds did not have lower survival probabilities than subordinates, which at first sight suggest there is little cost associated with reproduction itself. However, the assessment of status was based on observations only. A previous study showed that 44% of subordinate females are joint nesting each year [77], therefore they may also suffer any cost of reproduction. Furthermore, subordinates may have been investing considerable effort in helping. Unfortunately, we could not differentiate helper and non-helper survival as the minor breeding peak was monitored in a few years only, drastically reducing our sample size with respect to knowledge about whether a bird has helped or not. Finally, it might be that heterogeneity in quality between individuals, or condition dependence, allows certain individuals to reproduce without bearing the cost of reduced survival [78].
